# Supplementary figures and images for: Melatonin attenuates liver ischemia-reperfusion injury via inhibiting the PGAM5-mPTP pathway
Source: PLoS One. 2024 Oct 29;19(10):e0312853. doi: 10.1371/journal.pone.0312853 (PMC11521291; doi:10.1371/journal.pone.0312853)

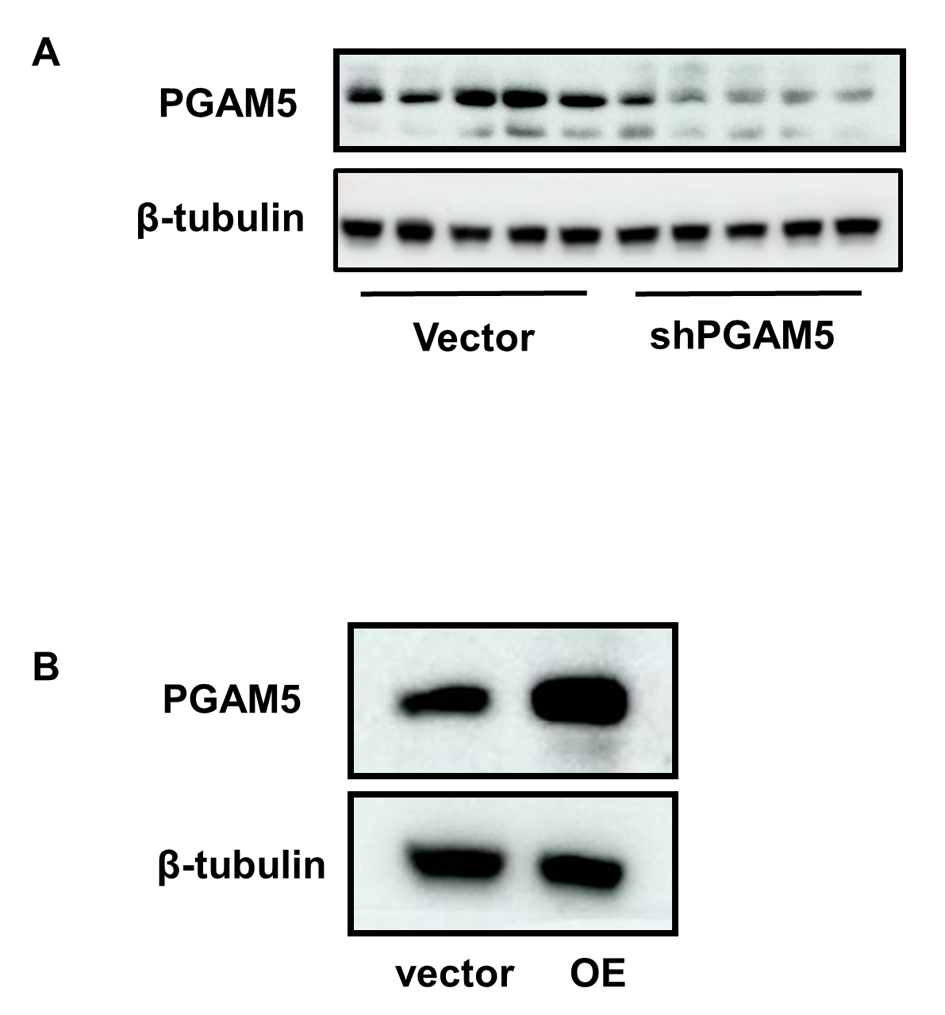

Supplement: S1 Fig — (A) Expression of PGAM5 in liver tissues from Vector and shPGAM5 mice was analyzed by western blotting. (B) the overexpression efficiency of PGAM5 in AML12 cells was verified by western blotting. (TIF) [file pone.0312853.s001.tif]
