# Supplementary material for: Melatonin attenuates liver ischemia-reperfusion injury via inhibiting the PGAM5-mPTP pathway
Source: PLoS One. 2024 Oct 29;19(10):e0312853. doi: 10.1371/journal.pone.0312853 (PMC11521291; doi:10.1371/journal.pone.0312853)

Panel A represent the western blot data shown in Figure 2

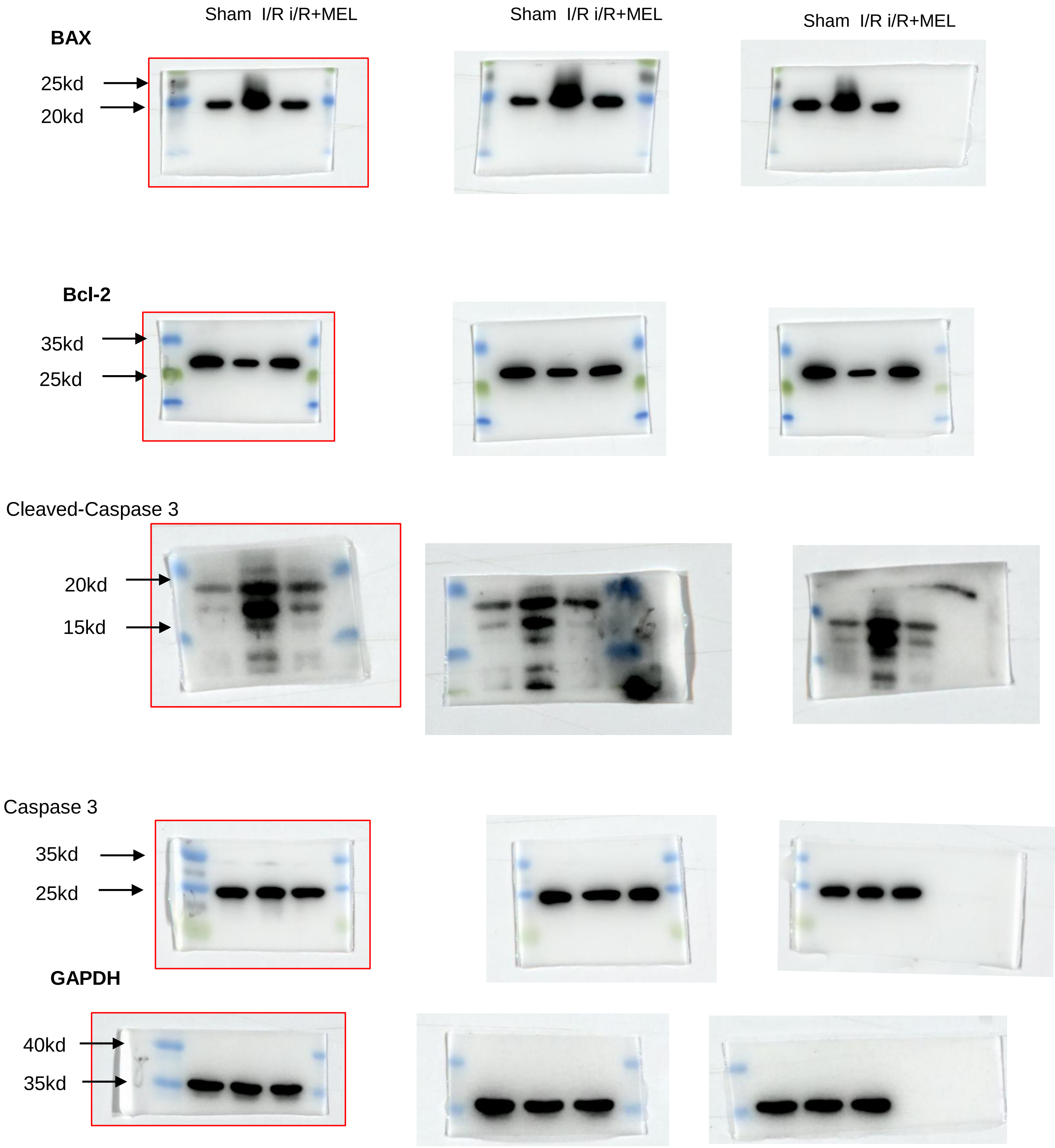

Panel A represent the western blot data shown in Figure 2

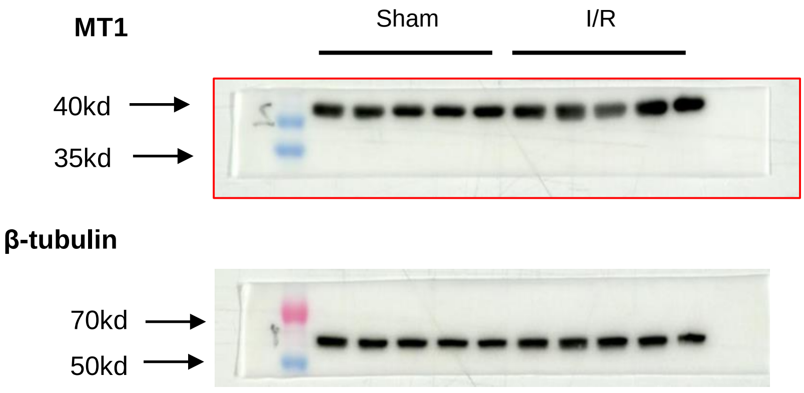

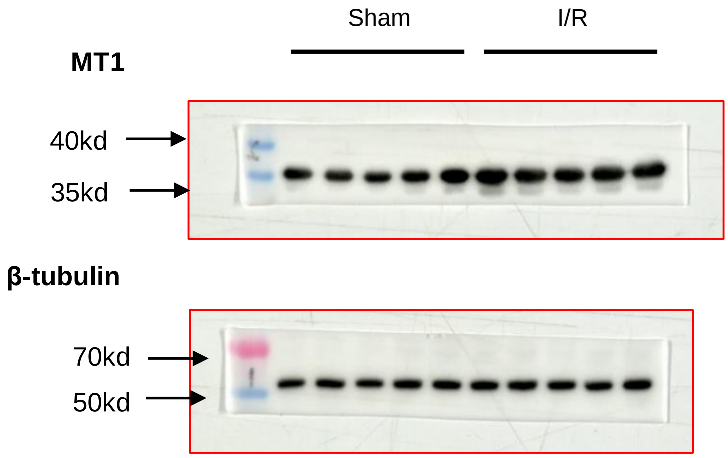

Panel B represent the western blot data shown in Figure 4

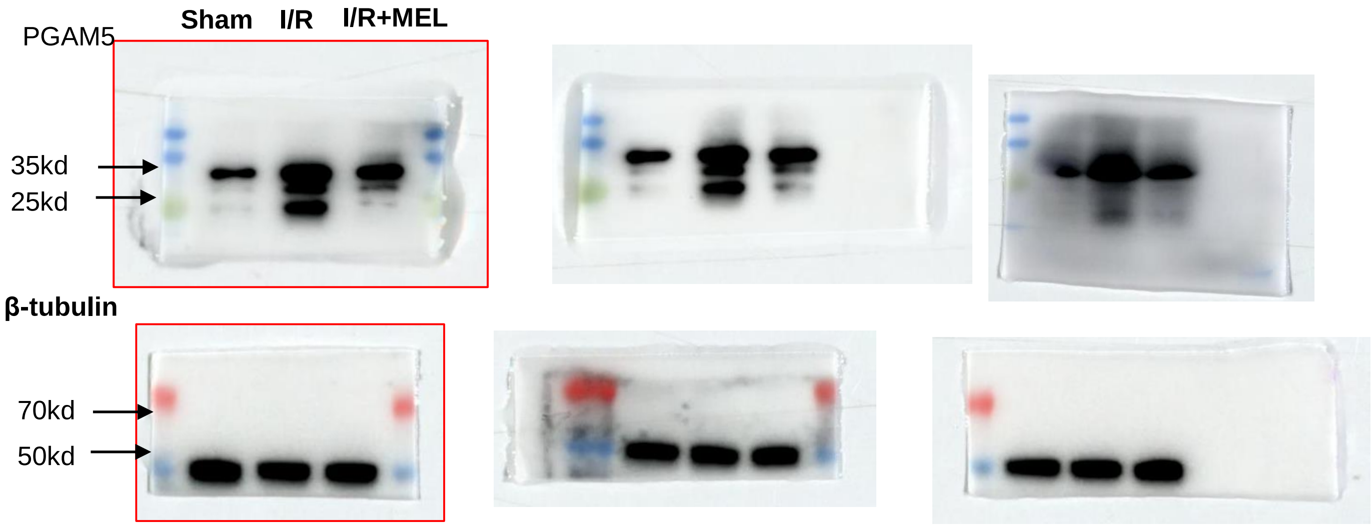

Panel E represent the western blot data shown in Figure s1

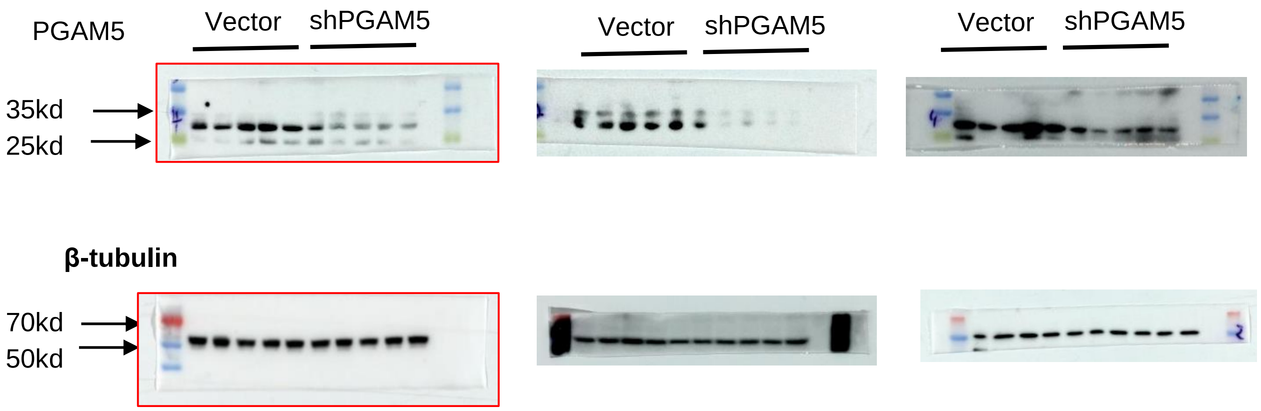

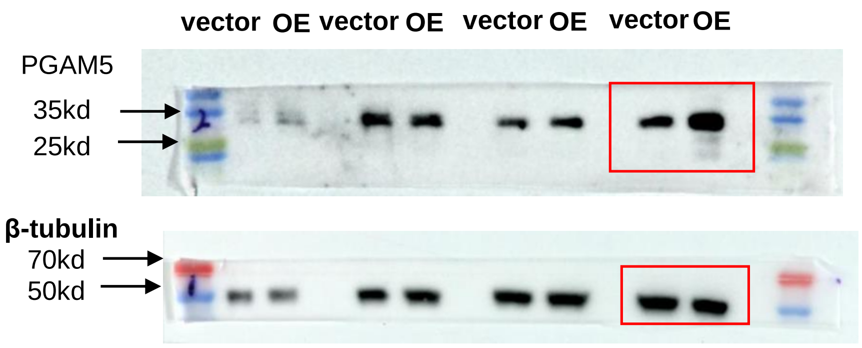

The protein marker used in the experiment was brought from Epizyme Biotech (http://www.epizyme.cn/) The catalogue number of protein marker is WJ103. Protein marker indicator band is shown in the figure below

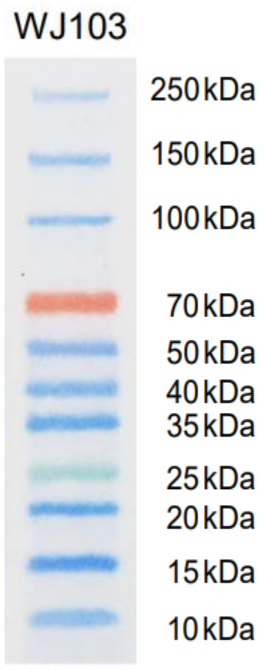

Supplement: S1 Raw images — (PDF) [file pone.0312853.s003.pdf]
