# Supplementary material for: Melatonin attenuates liver ischemia-reperfusion injury via inhibiting the PGAM5-mPTP pathway
Source: PLoS One. 2024 Oct 29;19(10):e0312853. doi: 10.1371/journal.pone.0312853 (PMC11521291; doi:10.1371/journal.pone.0312853)
Supplement: Attachment — Submitted filename: Review report PONE-D-24-18789.docx [file pone.0312853.s004.docx]

**Review report**

Ref. to the manuscript titled" *Melatonin attenuates liver ischemia-reperfusion injury via inhibiting the PGAM5-mPTP pathway*" with manuscript ID PONE-D-24-18786.

Overview:

The manuscript investigates the role of melatonin in attenuating liver ischemia-reperfusion (I/R) injury and the underlying mechanisms. The authors found that melatonin could alleviate liver I/R injury by inhibiting the expression of PGAM5, a key regulator of mitochondrial function. Mechanistically, melatonin blocked PGAM5-mediated mitochondrial permeability transition pore (mPTP) opening, thereby maintaining mitochondrial function and preventing hepatocyte death.

The study is well-designed and utilizes both in vivo and in vitro models to comprehensively investigate the protective effects of melatonin and the involvement of the PGAM5-mPTP axis. The data presented are convincing and support the authors' conclusions. The experiments are well-executed, and the results are clearly presented. The manuscript is generally well-written, with a clear flow and logical organization. The supplementary information is also relevant and supports the main findings. However, the quality of the figures needs improvement.

Concerns:

\# Introduction

- PGAM5 (phosphoglycerate mutase 5) should be defined in the introduction.
- The results of recent relevant studies [1-5] have not been included in both the introduction and discussion sections. The introduction and discussion should be substantially revised to incorporate these findings.

#Methods

- The in vivo study duration is not well stated. The sentence "Following the experiment, all mice were euthanized..." does not clearly indicate the study duration.
- It is unclear why a group of IR+ShPGAM5+Melatonin was not included.
- The terminology should be consistent, using "concentration" for in vitro studies and "dose" for in vivo studies.
- It would be better to separate the in vitro and in vivo study methods, presenting the in vitro methods first, followed by the in vivo methods.
- Clarify whether the western blot and real-time PCR analyses were performed on cell samples or animal samples, and present the methods in the appropriate order.
- The abbreviation "mPTP" (mitochondrial permeability transition pore) should be defined upon first use.

#Results

- Specify the data expression format (mean±SD or mean±SEM).
- In the first paragraph of the results section, the phrase "in the same vein" should be revised.
- The duration of cell hypoxia induction should be indicated.
- The sentence "high dose melatonin produced hepatoprotection" should be rewritten.
- The descriptions of the results shown in Figure 4 are inaccurate and should be corrected.
- The quality of the immunofluorescence images in Figure 4 and other figures should be improved.

#Discussion

The discussion effectively summarizes the key findings and places them in the context of the existing literature. The proposed mechanism is clearly explained and supported by the experimental data. The introduction should also include an explanation of PGAM5, similar to the discussion.

- In the last paragraph of the discussion, the format of the references should be corrected.

1- Yang M, Wang Z, Xie J, Reyad-ul-Ferdous M, Li S, Song Y. Cyclophilin D as a potential therapeutic target of liver ischemia/reperfusion injury by mediating crosstalk between apoptosis and autophagy. Chronic Diseases and Translational Medicine. 2023 Sep 25;9(03):238-49.
2- Mohamed DZ, El AE, Sokar SS, Shebl AM, Abu-Risha SE. Targeting autophagy to modulate hepatic ischemia/reperfusion injury: A comparative study between octreotide and melatonin as autophagy modulators through AMPK/PI3K/AKT/mTOR/ULK1 and Keap1/Nrf2 signaling pathways in rats. European Journal of Pharmacology. 2021 Apr 15;897:173920.
3- El AE, Sokar SS, Shebl AM, Mohamed DZ, Abu-Risha SE. Octreotide and melatonin alleviate inflammasome-induced pyroptosis through inhibition of TLR4-NF-κB-NLRP3 pathway in hepatic ischemia/reperfusion injury. Toxicology and Applied Pharmacology. 2021 Jan 1;410:115340.
4- Gao Y, Li ZT, Jin L, Lin J, Fan ZL, Zeng Z, Huang HF. Melatonin attenuates hepatic ischemia-reperfusion injury in rats by inhibiting NF-κB signaling pathway. Hepatobiliary & Pancreatic Diseases International. 2021 Dec 1;20(6):551-60.
5- Mao B, Yuan W, Wu F, Yan Y, Wang B. Autophagy in hepatic ischemia–reperfusion injury. Cell death discovery. 2023 Apr 5;9(1):115.
