# Supplementary material for: Melatonin attenuates liver ischemia-reperfusion injury via inhibiting the PGAM5-mPTP pathway
Source: PLoS One. 2024 Oct 29;19(10):e0312853. doi: 10.1371/journal.pone.0312853 (PMC11521291; doi:10.1371/journal.pone.0312853)
Supplement: Attachment — Submitted filename: Response to editor and Reviewers.docx [file pone.0312853.s005.docx]

**Response to Reviewers' Comments**

**Melatonin attenuates liver ischemia-reperfusion injury via inhibiting the PGAM5-mPTP pathway （**PONE-D-24-18786）

1. It appears that your ORCiD iD has not been validated in your Editorial Manager account and we are unable to proceed until that step is complete.

To validate your ORCiD iD in Editorial Manager, please follow the steps below:

a. In your Editorial Manager account, please go to ‘Update my Information’ (in the upper left-hand corner of the main menu), and click on the Fetch/Validate link next to the ORCiD field.

b. This link will take you to the ORCiD site and allow you to create a new iD or authenticate a pre-existing iD in Editorial Manager.

For additional instructions, please watch the following video for a step-by-step demonstration: https://www.youtube.com/watch?v=_xcclfuvtxQ

**Explanation**

Thanks very much! We have already validated our ORCiD id.

2. Your ethics statement should only appear in the Methods section of your manuscript. If your ethics statement is written in any section besides the Methods, please delete it from any other section.

**Explanation**

Thanks very much! We have already revised it.

3. We note your current Data Availability statement is: "All relevant data are within the manuscript and its Supporting Information files."

However, in your manuscript your data availability stated as follows: "The datasets used and/or analyzed during the current study available from the corresponding author on reasonable request."

Please update your Data Availability statement accordingly.

**Explanation**

Thanks very much! We have already revised it.

4. To comply with PLOS ONE submissions requirements, please provide the following information in the Methods section of the manuscript.

If anesthesia, euthanasia, or any kind of animal sacrifice is part of the study, please include briefly in your statement which substances and/or methods were applied.

**Explanation**

Thanks very much! We have already revised it.

**Reviewer Comments:**

**Reviewer #1:** The study entitled “Melatonin attenuates liver ischemia-reperfusion injury via inhibiting the PGAM5-mPTP pathway” has tried to examine the role of PGAM5 in hepatic IR injury and its involvement in the melatonin-mediated hepatic protection. The authors have used both in vivo and in vitro models to prove the hypothesis. Although the melatonin-induced protection in liver I/R injury has been well established via HO-1 and inhibiting the NF-κB signaling pathway, the role of PGAM-5 has been explored for the first time in this manuscript. In order to do so, the authors have overexpressed and silenced PGAM5 and extensive work has been done with western blots, immunofluorescence and immunohistochemistry analysis to support the protective effects of melatonin and PGAM5 silencing. The paper contains important and novel findings and should have implications in clinical settings dealing with diagnosing and treating liver injury。

**Explanation**

Thanks very much!

**Reviewer #2: # Introduction**

- PGAM5 (phosphoglycerate mutase 5) should be defined in the introduction.

**Explanation**

Thanks very much! We have already defined it in the revised manuscript.

- The results of recent relevant studies [1-5] have not been included in both the introduction and discussion sections. The introduction and discussion should be substantially revised to incorporate these findings.

**Explanation**

Thanks for your suggestion. We have already revised it.

#Methods

- The in vivo study duration is not well stated. The sentence "Following the experiment, all mice were euthanized..." does not clearly indicate the study duration.

**Explanation**

Thanks for your suggestion. We have already revised it.

- It is unclear why a group of IR+ShPGAM5+Melatonin was not included.

**Explanation**

Thanks for your suggestion. Our previous results have shown that melatonin can inhibit PGAM5 expression. Therefore, we thought that if silencing PGAM5 could reduce mouse liver IRI, then Melatonin can work by inhibiting PGAM5. Moreover, our preliminary experiments also found that melatonin had no effect in PGAM5-silenced mice (data not shown). In the recovery experiment, we selected aml12 cells and transfected PGAM5 with lentivirus to see whether overexpression of PGAM5 could neutralize the role of melatonin. We think that these two positive and negative experiments can clarify our hypothesis. Therefore, we did not set up the IR+ShPGAM5+Melatonin group.

- The terminology should be consistent, using "concentration" for in vitro studies and "dose" for in vivo studies.

**Explanation**

Thanks for your suggestion. We have already revised it.

- It would be better to separate the in vitro and in vivo study methods, presenting the in vitro methods first, followed by the in vivo methods.

**Explanation**

Thanks for your suggestion. We have already revised it.

- Clarify whether the western blot and real-time PCR analyses were performed on cell samples or animal samples, and present the methods in the appropriate order.

**Explanation**

Thanks for your suggestion. We have already revised it.

- The abbreviation "mPTP" (mitochondrial permeability transition pore) should be defined upon first use.

**Explanation**

Thanks for your suggestion. We have already revised it.

#Results

- Specify the data expression format (mean±SD or mean±SEM).

**Explanation**

Thanks for your suggestion. We have already revised it.

- In the first paragraph of the results section, the phrase "in the same vein" should be revised.

**Explanation**

Thanks for your suggestion. We have already revised it.

- The duration of cell hypoxia induction should be indicated.

**Explanation**

Thanks for your suggestion. We have already indicated the duration of cell hypoxia in the revised manuscript.

- The sentence "high dose melatonin produced hepatoprotection" should be rewritten.

**Explanation**

Thanks for your suggestion. We have already revised it.

- The descriptions of the results shown in Figure 4 are inaccurate and should be corrected.

**Explanation**

Thanks for your suggestion. We have already revised it.

- The quality of the immunofluorescence images in Figure 4 and other figures should be improved.

**Explanation**

Thanks for your suggestion. The figures have been improved in the revised manuscript.

#Discussion

The discussion effectively summarizes the key findings and places them in the context of the existing literature. The proposed mechanism is clearly explained and supported by the experimental data. The introduction should also include an explanation of PGAM5, similar to the discussion.

**Explanation**

Thanks for your suggestion. We have already revised it.

- In the last paragraph of the discussion, the format of the references should be corrected.

**Explanation**

Thanks for your suggestion. We have already revised it.

1- Yang M, Wang Z, Xie J, Reyad-ul-Ferdous M, Li S, Song Y. Cyclophilin D as a potential therapeutic target of liver ischemia/reperfusion injury by mediating crosstalk between apoptosis and autophagy. Chronic Diseases and Translational Medicine. 2023 Sep 25;9(03):238-49.

2- Mohamed DZ, El AE, Sokar SS, Shebl AM, Abu-Risha SE. Targeting autophagy to modulate hepatic ischemia/reperfusion injury: A comparative study between octreotide and melatonin as autophagy modulators through AMPK/PI3K/AKT/mTOR/ULK1 and Keap1/Nrf2 signaling pathways in rats. European Journal of Pharmacology. 2021 Apr 15;897:173920.

3- El AE, Sokar SS, Shebl AM, Mohamed DZ, Abu-Risha SE. Octreotide and melatonin alleviate inflammasome-induced pyroptosis through inhibition of TLR4-NF-κB-NLRP3 pathway in hepatic ischemia/reperfusion injury. Toxicology and Applied Pharmacology. 2021 Jan 1;410:115340.

4- Gao Y, Li ZT, Jin L, Lin J, Fan ZL, Zeng Z, Huang HF. Melatonin attenuates hepatic ischemia-reperfusion injury in rats by inhibiting NF-κB signaling pathway. Hepatobiliary & Pancreatic Diseases International. 2021 Dec 1;20(6):551-60.

5- Mao B, Yuan W, Wu F, Yan Y, Wang B. Autophagy in hepatic ischemia–reperfusion injury. Cell death discovery. 2023 Apr 5;9(1):115.

Reviewer #3: The manuscript by Tang et al. shows that melatonin inhibits liver injury by reducing the activity PGAM5. I have two major comments:

1) You do not mention and cite your recent paper in Scientific Reports (PMID: 38609411) on PGAM5 and liver injury. Please discuss how the new findings relate with the published ones.

**Explanation**

Thanks for your suggestion. When we discovered the role of PGAM5 in liver I/R injury in mice, we wondered if we could find a small-molecule inhibitor that could inhibit PGAM5. Our review of the literature found that Melatonin protects mitochondrial function and reduces myocardial ischemia-reperfusion damage. Therefore, we speculate that melatonin may reduce liver I/R injury by regulating hepatocyte mitochondrial function through PGAM5. Since the two projects were completed almost simultaneously, at the time of writing this article, the other one had not yet been published. Therefore, we did not cite this article.

2) The most important question is the mechanism by which melatonin exerts its effects on hepatocytes. Do they have receptors and can you inhibit the effects by blocking them? What are the intracellular signalling pathways?

**Explanation**

Thanks for your suggestion. The literature has confirmed that melatonin activates two high-affinity G protein-coupled receptors, termed MT1 and MT2, to exert beneficial actions. Therefore, we did not further investigate this. In this study, we mainly explored how melatonin, after entering cells, regulates mitochondrial function and alleviates liver IRI in mice.

1.Liu J, Clough SJ, Hutchinson AJ, Adamah-Biassi EB, Popovska-Gorevski M, Dubocovich ML. MT1 and MT2 Melatonin Receptors: A Therapeutic Perspective. Annu Rev Pharmacol Toxicol. 2016;56:361-83. doi: 10.1146/annurev-pharmtox-010814-124742. Epub 2015 Oct 23. PMID: 26514204; PMCID: PMC5091650.

2. Kinker GS, Ostrowski LH, Ribeiro PAC, Chanoch R, Muxel SM, Tirosh I, Spadoni G, Rivara S, Martins VR, Santos TG, Markus RP, Fernandes PACM. MT1 and MT2 melatonin receptors play opposite roles in brain cancer progression. J Mol Med (Berl). 2021 Feb;99(2):289-301. doi: 10.1007/s00109-020-02023-5. Epub 2021 Jan 3. PMID: 33392634.

3. González-Arto M, Aguilar D, Gaspar-Torrubia E, Gallego M, Carvajal-Serna M, Herrera-Marcos LV, Serrano-Blesa E, Hamilton TR, Pérez-Pé R, Muiño-Blanco T, Cebrián-Pérez JA, Casao A. Melatonin MT₁ and MT₂ Receptors in the Ram Reproductive Tract. Int J Mol Sci. 2017 Mar 19;18(3):662. doi: 10.3390/ijms18030662. PMID: 28335493; PMCID: PMC5372674.

Minor:

3) Which tissue is shown in Fig. S1A? What happens in other organs of the mouse, is PGAM5 downregulated in the whole body? The legend for this figure needs to be more detailed.

**Explanation**

Thanks for your suggestion. We have already revised the legend. Liver tissues form mice were shown in Fig.S1A. :Proteins were isolated from livers. Due to financial constraints, we did not choose to specifically silence PGAM5 in mouse liver. Additionally, we detected PGAM5 expression in mouse kidneys and found that PGAM5 was also silenced in the kidneys (data not shown).

4) Please check the axis labels in figure 5G: Death cells ...

**Explanation**

Thanks for your suggestion. We have already revised it.
