# Supplementary material for: Melatonin attenuates liver ischemia-reperfusion injury via inhibiting the PGAM5-mPTP pathway
Source: PLoS One. 2024 Oct 29;19(10):e0312853. doi: 10.1371/journal.pone.0312853 (PMC11521291; doi:10.1371/journal.pone.0312853)
Supplement: Attachment — Submitted filename: Response to Reviewers .docx [file pone.0312853.s006.docx]

**Response to Reviewers' Comments**

**Melatonin attenuates liver ischemia-reperfusion injury via inhibiting the PGAM5-mPTP pathway （**PONE-D-24-18786）

Reviewer #3: The authors have not addressed my two major comments:

1) The Scientific Reports paper needs to be cited and the whole manuscript needs to be rewritten telling what is already shown in the published paper and what is new in the current one.

Explanation

Thanks very much! The Scientific Reports have been cited. We had two innovations in this study: 1, we identified the role of PGAM5 in melatonin-reducing I/R injury in the liver of mice. 2, Mechanistically, this study extends previous research. There is a certain relationship between the opening of MPTP and mitochondrial fission. The opening of MPTP can trigger the release of calcium ions within the mitochondria and lead to mitochondrial dysfunction, thereby affecting the fission and fusion process of mitochondria. During mitochondrial fission, the opening of MPTP may cause damage to the inner mitochondrial membrane and metabolic instability, which may exacerbate cellular stress and death. Combining our study with this, we can hypothesize that PGAM5-mediated MPTP-opening may be a prerequisite for activating Drp1 phosphorylation and inducing mitochondrial fission. These contents have been integrated into the Instruction and Discussion.

2) The authors need at least to speculate how melatonin can enter the cell and with which receptors it may interact there. Anyhow their data could also be explained by interacting of melatonin with its receptors on the cell surface. There is no evidence that it enters the cell. That's why experiments need to be done on cells with M1 and M2 receptor antagonists to exclude these pathways.

Explanation

Thanks for your suggestion. We have made the changes according to your suggestion. To investigate which melatonin receptor plays a role, both MT1 and MT2 subtypes were detected in the liver of mice by western blot. Notably, MT2 expression significantly elevated after liver I/R injury, whereas MT1 level remained unaltered (Figure 2 F). Afterward, we used 4P-PDOT to block MT2 receptors. As shown in Figure 2 G and H, 4-P-PDOT negated the effect of melatonin. Thank you again for your proposal.
