# Supplementary material for: Melatonin attenuates liver ischemia-reperfusion injury via inhibiting the PGAM5-mPTP pathway
Source: PLoS One. 2024 Oct 29;19(10):e0312853. doi: 10.1371/journal.pone.0312853 (PMC11521291; doi:10.1371/journal.pone.0312853)
Supplement: Attachment — Submitted filename: Response to Reviewers .docx [file pone.0312853.s007.docx]

**Response to Reviewers' Comments**

**Melatonin attenuates liver ischemia-reperfusion injury via inhibiting the PGAM5-mPTP pathway （**PONE-D-24-18786）

1) Antibodies agains GPCRs are notoriously unspecific, please provide the details which antibodies you used for MT1 and MT2 and evidence that they are specific.

Explanation

Thanks for your suggestion. The details of antibodies have been added to the methods section.

2) Receptors do not need to be upregulated to be involved in physiological actions of ligands. Thus Fig. 2F does not exclude MT1 as mediator of melatonin actions. Please mitigate your conclusion that only MT2 is involved.

Explanation

Thanks for your suggestion. We have already revised it.
